# Construction of competitive endogenous RNA network reveals regulatory role of long non-coding RNAs in intracranial aneurysm

**DOI:** 10.1186/s12868-021-00622-7

**Published:** 2021-03-09

**Authors:** Yuan-Bo Pan, Jianan Lu, Biao Yang, Cameron Lenahan, Jianmin Zhang, Anwen Shao

**Affiliations:** 1 Department of Neurosurgery, School of Medicine, Second Affiliated Hospital, Zhejiang University, NO.88 Jiefang Rd, Hangzhou, 310009 Zhejiang China; 2 Department of Neurosurgery, Huashan Hospital of Fudan University, Shanghai, China; 3 Burrell College of Osteopathic Medicine, Las Cruces, NM 88003 USA; 4 Center for Neuroscience Research, School of Medicine, Loma Linda University, Loma Linda, CA 92324 USA; 5 Brain Research Institute, Zhejiang University, Hangzhou, Zhejiang China; 6 Collaborative Innovation Center for Brain Science, Zhejiang University, Hangzhou, Zhejiang China

**Keywords:** Bioinformatics analysis, Intracranial aneurysm, Competitive endogenous RNA, Long non-coding RNA, PI3K-Akt signaling pathway, PVT1, HOTAIR

## Abstract

**Background:**

Rupture of intracranial aneurysm (IA) is the main cause of devastating subarachnoid hemorrhage, which urges our understanding of the pathogenesis and regulatory mechanisms of IA. However, the regulatory roles of long non-coding RNAs (lncRNAs) in IA is less known.

**Results:**

We processed the raw SRR files of 12 superficial temporal artery (STA) samples and 6 IA samples to count files. Then the differentially expressed (DE) mRNAs, miRNAs, and lncRNAs between STAs and IAs were identified. The enrichment analyses were performed using DEmRNAs. Next, a lncRNA-miRNA-mRNA regulatory network was constructed using integrated bioinformatics analysis. In summary, 341 DElncRNAs, 234 DEmiRNAs, and 2914 DEmRNAs between the STA and IA. The lncRNA-miRNA-mRNA regulatory network of IA contains 91 nodes and 146 edges. The subnetwork of hub lncRNA PVT1 was extracted. The expression level of PVT1 was positively correlated with a majority of the mRNAs in its subnetwork. Moreover, we found that several mRNAs (CCND1, HIF1A, E2F1, CDKN1A, VEGFA, COL1A1 and COL5A2) in the PVT1 subnetwork served as essential components in the PI3K-Akt signaling pathway, and that some of the non-coding RNAs (ncRNAs) (PVT1, HOTAIR, hsa-miR-17, hsa-miR-142, hsa-miR-383 and hsa-miR-193b) interacted with these mRNAs.

**Conclusion:**

Our annotations noting ncRNA’s role in the pathway may uncover novel regulatory mechanisms of ncRNAs and mRNAs in IA. These findings provide significant insights into the lncRNA regulatory network in IA.

**Supplementary Information:**

The online version contains supplementary material available at 10.1186/s12868-021-00622-7.

## Background

Rupture of intracranial aneurysm (IA) is the main cause of subarachnoid hemorrhage (SAH), leading to exceedingly high mortality and morbidity [1, 2]. The prevalence of IA in the general population is reported to be 3.2% [1]. Approximately 85% of non-traumatic SAH is caused by IA rupture [3]. Moreover, although unruptured IAs (UIs) are typically asymptomatic, UIs are being detected more and more, and have become an important health issue [1]. Since surgical clipping and intravascular coiling are invasive procedures with potentially serious complications, the effective management of UIs remains a challenge [4]. A deeper understanding of the pathogenesis of IA helps to find more effective treatment for IA.

Recently, an increasing number of studies have focused on the competitive endogenous RNA (ceRNA) network’s impact in cardiac hypertrophy, type 2 diabetes mellitus, and various types of cancer [5–8]. The hypothesis of ceRNA described a molecular regulatory mechanism for post-transcriptional regulation. Several studies revealed that lncRNAs can function as microRNA (miRNA) sponges in a ceRNA regulatory network, and further regulate mRNA expression levels [9]. LncRNAs are non-coding RNA of greater than 200 nucleotides in length, which were reported to play a crucial role in the biological processes [10]. However, the potential role of the ceRNA regulatory network in IA formation remains unclear.

In this study, we identified differentially expressed lncRNAs, miRNAs, and mRNAs between STAs and IAs. Then, we developed a lncRNA-miRNA-mRNA regulatory network. Previous study found that knockdown of lncRNA PVT1 could reverse the murine angiotensin II-induced abdominal aortic aneurysm (AAA) associated alterations, including inhibition of vascular smooth muscle cell (VSMC) apoptosis and ECM disruption [11]. However, the regulatory mechanism of lncRNA PVT1 in development of IA was not clear. Thus, lncRNA PVT1 was selected for further analysis. The correlations between PVT1 and mRNAs in the PVT1 subnetwork were analyzed. After a functional enrichment analysis, we found that mRNAs in the PVT1 subnetwork were annotated into the PI3K-Akt signaling pathway. In addition, we found that several ncRNAs were connected with these mRNAs, and we annotated these ncRNAs to the PI3K-Akt signaling pathway, which may uncover novel regulatory mechanisms of ncRNAs and mRNAs in IA.

## Materials and methods

### Patients and RNA-seq data processing

The SRR files (SRR1819905–SRR1819922), including 12 superficial temporal artery (STA) samples and 6 intracranial aneurysm (IA) samples, were retrieved from GEO NCBI [12] for single ended data, which were converted to ‘fastq’ format data using sratoolkit (version 2.8.2). The reference genome of human (GRCh38.p13) was downloaded from Ensembl (https://asia.ensembl.org/). STAR (version 2.4.0i) [13], a highly efficient RNAseq alignment tool. This was used to align the ‘fastq’ format data with the human reference genome, using the custom default parameters. The SAM files from STAR alignment were converted to the BAM format by using SAMtools (version 1.3.1) [14]. The BAM files were converted to counts files by using HTSeq [15].We generated “.csv” files, each consisting of all gene counts for that particular sample. Furthermore, we combined all of the “.csv” files and obtained a single file with sample names depicted as columns, and gene names depicted as rows. Then, the counts files were normalized using the edgER package [16]. LncRNAs and mRNAs were annotated using the Ensembl database [16]. Regarding the microRNA data in GSE66240, the raw probe-level data was preprocessed through the robust multi-array average (RMA) algorithm in the Affy package [17]. For genes corresponding to multiple probes, we used the average probe value as the expression level [18]. The missing data in these gene expression matrices were imputed with the k-Nearest Neighbor (KNN) method (k = 10) [19]. As all the data were retrieved from the GEO database, the approval from the local Ethics Committee was not needed.

### Identification of DElncRNAs, DEmiRNAs and DEmRNAs

Counts files, containing expression of lncRNAs and mRNAs, were normalized by the edgER package. Using the limma package, DElncRNAs, DEmiRNAs, and DEmRNAs were identified. lncRNAs, miRNAs, and mRNAs with |Log_2_(fold change)|> 1 and adjusted P-values < 0.05 were considered differentially expressed lncRNAs, miRNAs, and mRNAs.

### Functional enrichment analysis

Both the Gene ontology (GO) enrichment and ﻿Kyoto Encyclopedia of Genes and Genomes (KEGG) pathway enrichment analyses were performed using the ﻿Database for Annotation, Visualization, and Integrated Discovery platform (DAVID 6.8, https://david.ncifcrf.gov/) [20]. DEmRNAs were utilized for GO and KEGG pathway enrichment analyses. The pathways and GO terms, with corrected P-values < 0.05 using the Benjamini method, were considered significant categories.

### Construction of a ceRNA regulatory network

**Fig. 1:**
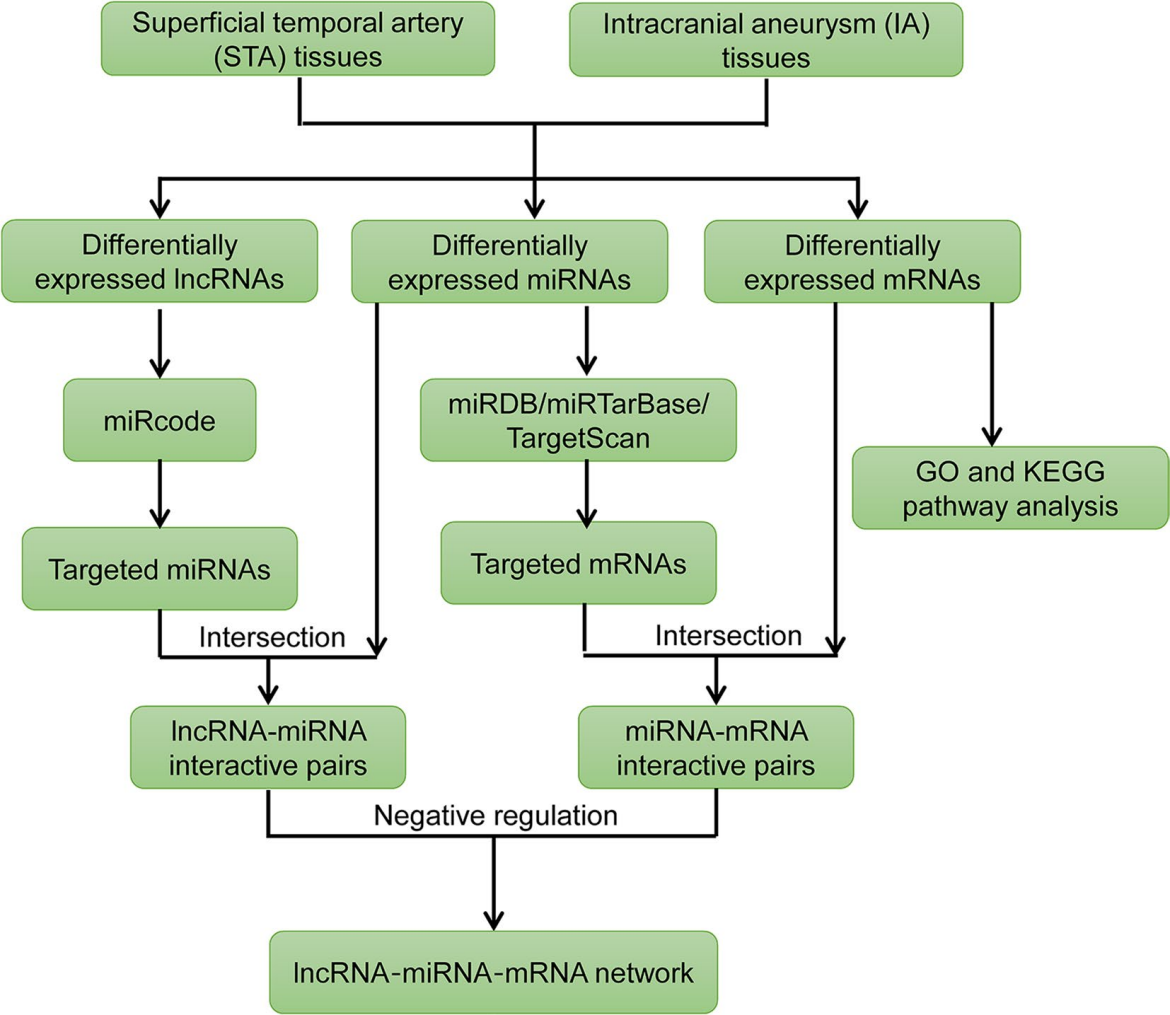
Flow chart of comprehensive bioinformatics analysis in the construction of lncRNA-miRNA-mRNA network

Figure 1 depicts the flow chart of the ceRNA network construction. Firstly, the lncRNA-miRNA potential interaction file (Highly conserved microRNA families) was downloaded from miRcode [21]. MiRcode provides a whole transcriptome human microRNA target prediction based on comprehensive GENCODE gene annotation, including > 10,000 long non-coding RNA genes. Secondly, DElncRNAs were put into the miRcode database and lncRNA-miRNA pairs were identified. Then, we eliminated miRNAs that expressed no difference between the STA and IA tissues. Thirdly, miRNA-targeted mRNAs were identified using three miRNA reference databases: miRDB, miRTarBase, and TargetScan, which contain predictive or experimental validated miRNA-targeted mRNAs [22–24]. Only mRNAs appearing in the three databases were defined as miRNA-targeted mRNAs for increasing the prediction reliability. Furthermore, targeted mRNAs that expressed no difference between STA and IA tissues were filtered out. The ceRNA network was constructed and viewed using Cytoscape (http://www.cytoscape.org/). The relationship between PVT1 and mRNA expression level was analyzed with Pearson correlation. The heatmaps based on DEmRNAs, DEmiRNAs and DElncRNAs included in the ceRNA regulatory network were generated by “pheatmap” package.

### Statistical analysis

GraphPad Prism (version 6.0, GraphPad Software, San Diego, CA, USA) and R language (3.4.0) were used for statistical analysis. A P-value < 0.05 was considered statistically significant.

## Results

### Differentially expressed RNAs in IA

Compared with the superficial temporal artery (STA) tissues, a total 2914 differentially expressed mRNA, 234 miRNA and 341 lncRNA were identified in intracranial aneurysm tissues. Of these, 201 (58.8%) lncRNAs, 1.807 (62%) mRNAs and 10 (4.3%) miRNAs were upregulated, whereas 141 (41.2%) lncRNAs, 1.107 (38%) mRNAs, and 224 (95.7%) miRNAs were downregulated in the tissue of intracranial aneurysm, when compared with STA tissues.

### Functional enrichment analysis of DEmRNAs

**Fig. 2:**
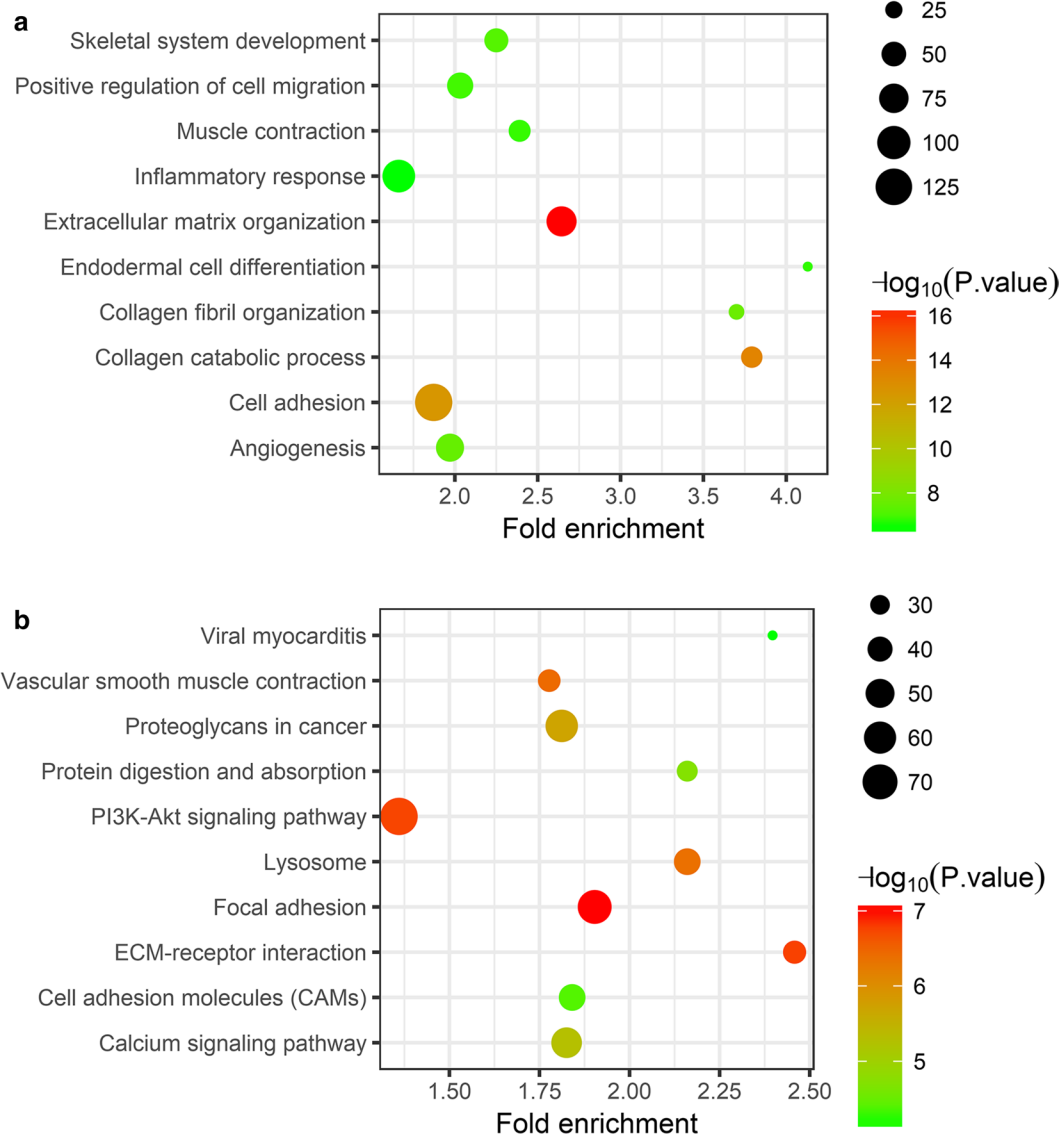
Functional enrichment analysis of differentially expressed mRNAs. **a** The top 10 enriched terms in the GO biological process analysis. **b** The top 10 enriched pathways in the KEGG pathway analysis. The size of circles represents the number of genes enriched in corresponding terms

GO enrichment and KEGG pathway analyses were performed to explore the potential functions of the 2.914 differentially expressed mRNAs. In the GO enrichment analysis, a total of 378 enriched GO terms have been identified in the Biological Process (BP). And the top 10 significantly enriched terms are shown in Fig. 2a. Moreover, the DEmRNAs were primarily enriched in collagen and extracellular matrix-related BPs, such as “extracellular matrix organization”, “collagen catabolic process”, “collagen fibril organization”, and “cell adhesion”. Moreover, we also found that these DEmRNAs were also enriched in “angiogenesis”, “skeletal system development”, “muscle contraction”, and “inflammatory response”, which indicates that these BPs may play roles in IA formation. In addition, a total 53 enriched pathways were identified after the KEGG pathway analysis. The top 10 enriched pathways are shown in Fig. 2b. And “vascular smooth muscle contraction” is closely associated with IA formation in these pathways. Furthermore, the enriched “focal adhesion”, “ECM-receptor interaction”, and “PI3K-Akt signaling pathway” indicated that the PI3K-Akt signaling pathway may play an important role in IA, which was further analyzed in this study.

### Construction of a ceRNA regulatory network in IA

**Fig. 3:**
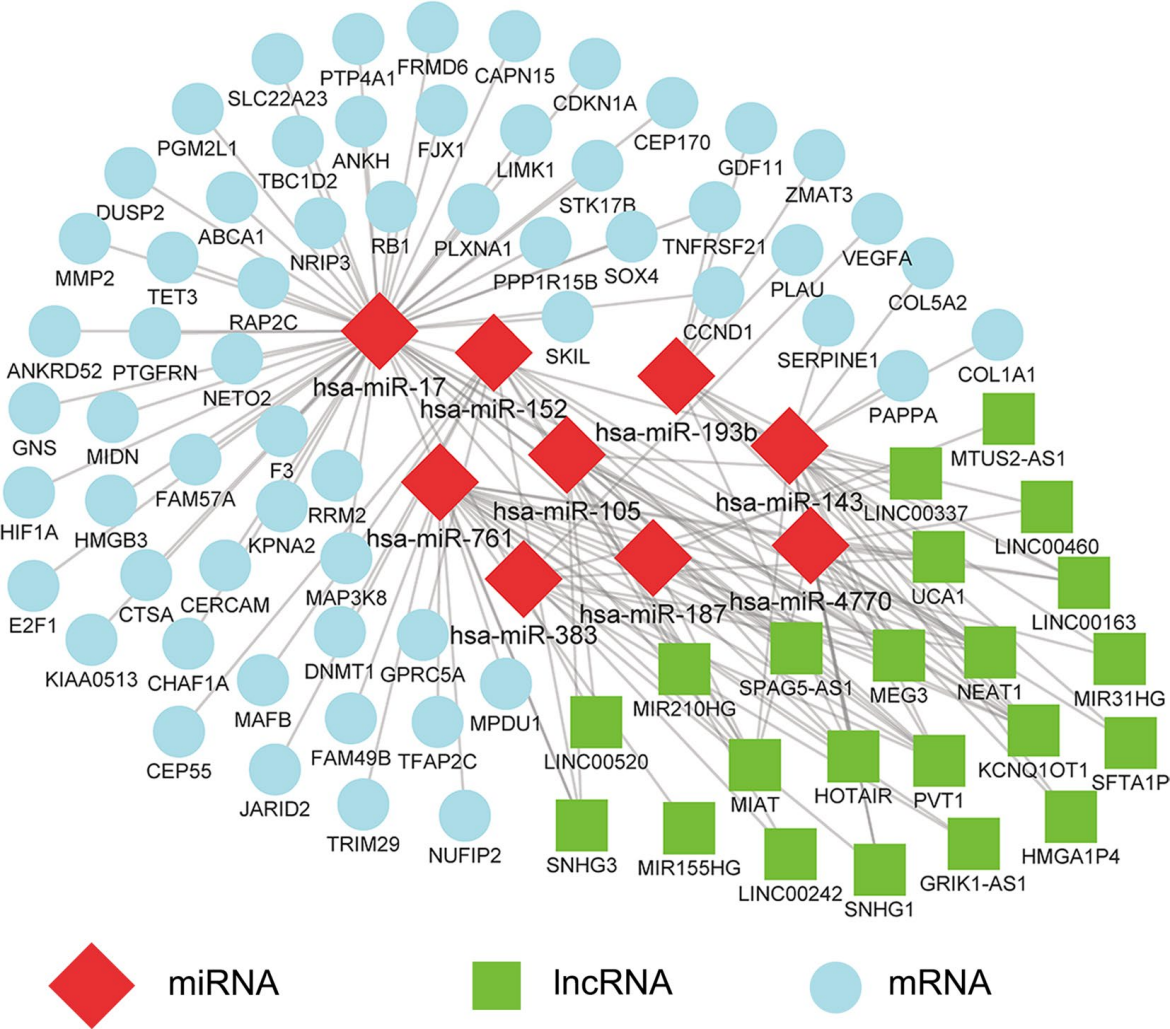
ceRNA network related to IA. Nodes in skyblue, red, and green represented mRNAs, miRNAs and lncRNAs, respectively

**Fig. 4:**
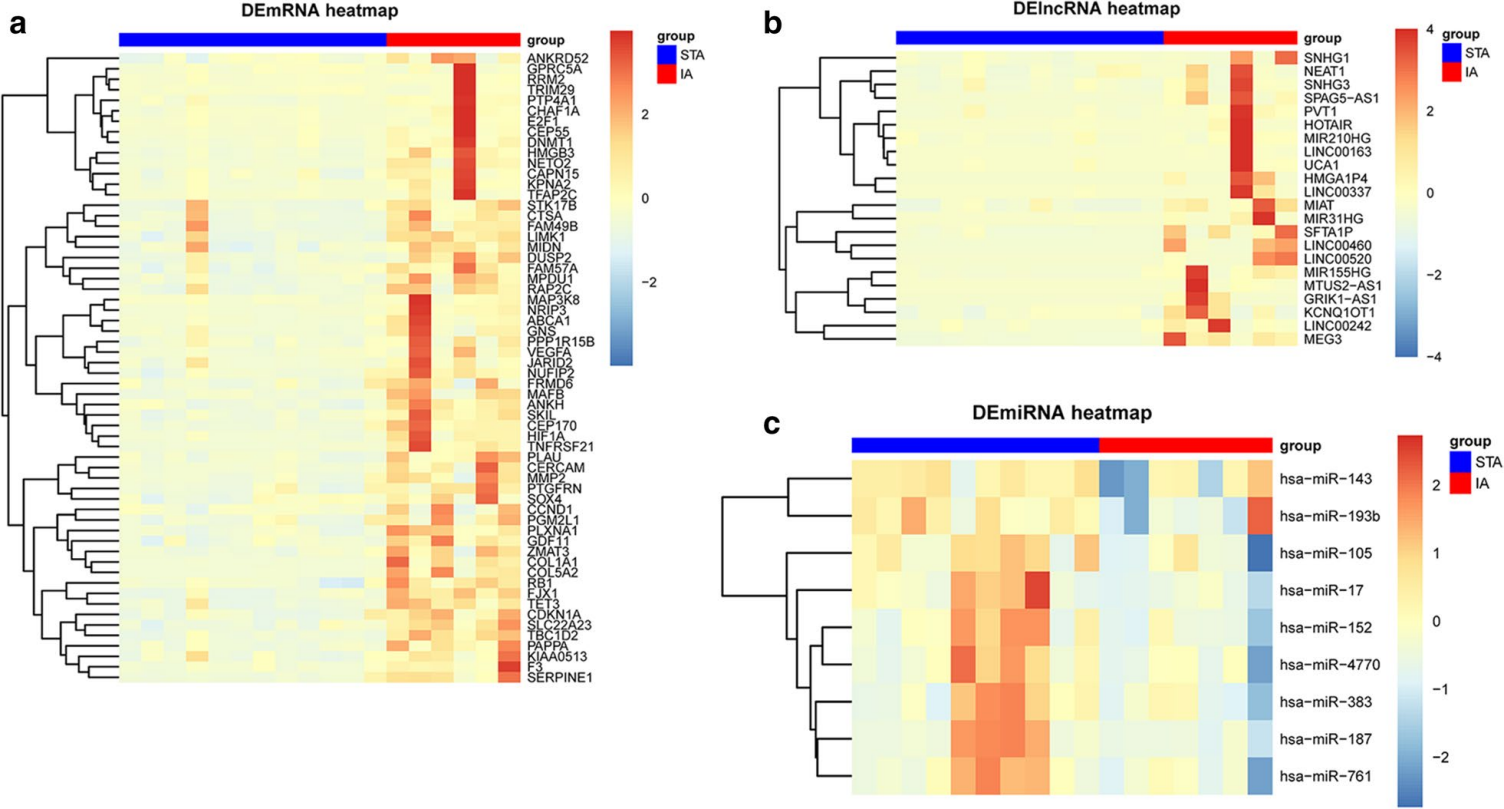
Hierarchical cluster heatmaps of differentially expressed mRNAs (**a**), lncRNAs (**b**) and miRNAs (**c**) included in the ceRNA network. Each row represents an RNA, and each column represents a sample. Red indicates relatively high expression, and blue indicates relatively low expression

**Fig. 5:**
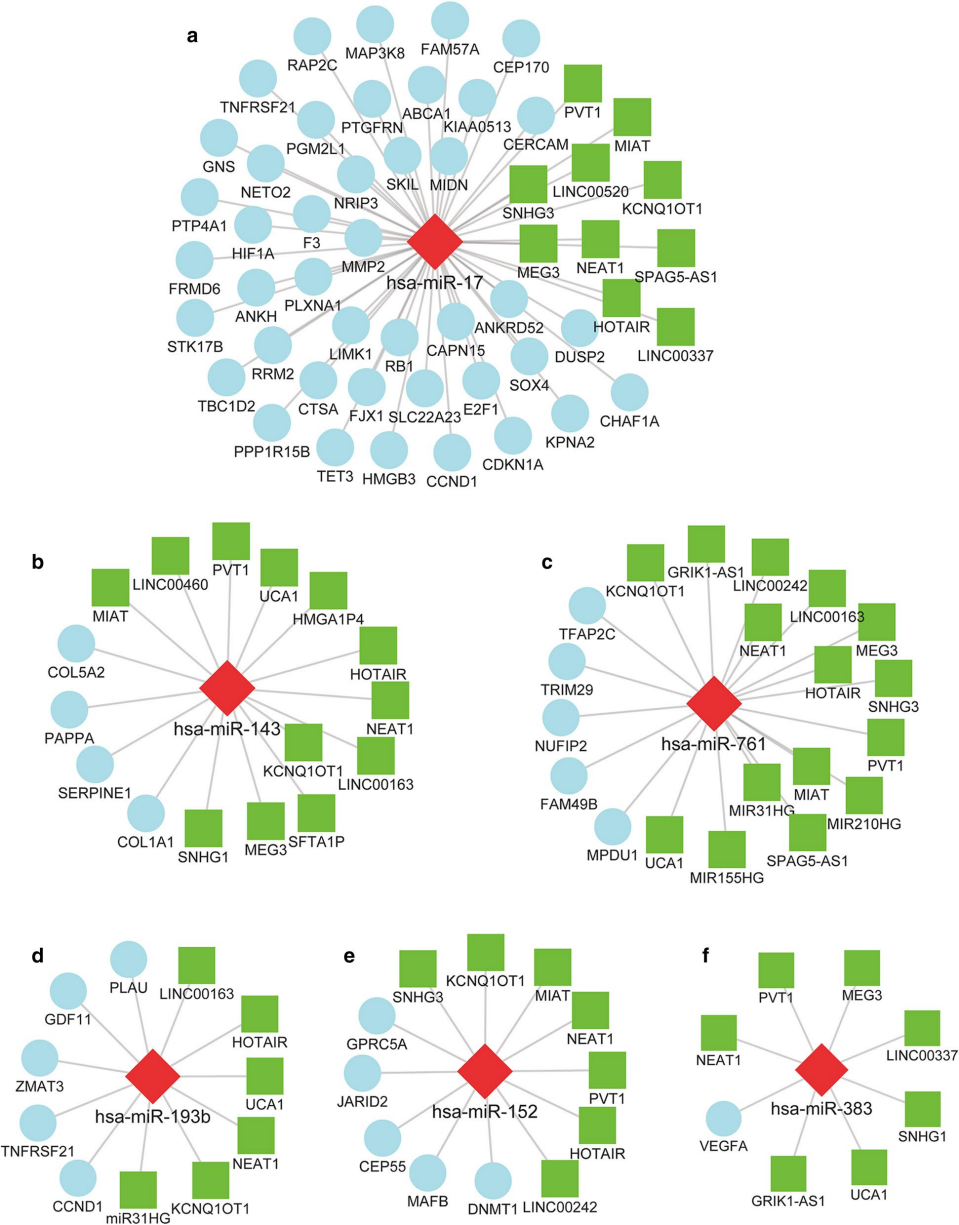
The subnetwork of each miRNA, including hsa-miR-17 (**a**), hsa-miR-143 (**b**), hsa-miR-761 (**c**), hsa-miR-193b (**d**), hsa-miR-152 (**e**) and hsa-miR-383 (**f**)

A lncRNA‐miRNA‐mRNA regulatory network of IA was constructed (Fig. 3) for exploring the regulatory mechanism of IA. The mRNA-miRNA and lncRNA‐miRNA relationship pairs (Additional file 1: Table S1, Additional file 2: Table S2) were combined into the ceRNA network, following a negative regulation pattern. The positively co-expressed mRNA-miRNA and lncRNA‐miRNA pairs were excluded. Finally, the network was constructed with 91 nodes (60 mRNAs, 9 miRNAs and 22 lncRNAs) and 146 edges. The degree of a node is defined as the total number of edges connecting it in the network, with an increased degree indicating that the node is highly connected, and likely to be a hub gene. The heatmaps based on DEmRNAs, DEmiRNAs and DElncRNAs included in the ceRNA regulatory network were shown in Fig. 4. Among the DEmiRNAs in the network, hsa-miR-17 has the highest degree (degree = 52), indicating that 52 nodes directly connect to it (Fig. 5). Moreover, other DEmiRNAs that connect lncRNAs and mRNAs in the network are also shown (Fig. 5). The highest degree DElncRNAs were PVT1, NEAT1 and KCNQ1OT1 (degree = 8).

### The components of PVT1 subnetwork

**Fig. 6:**
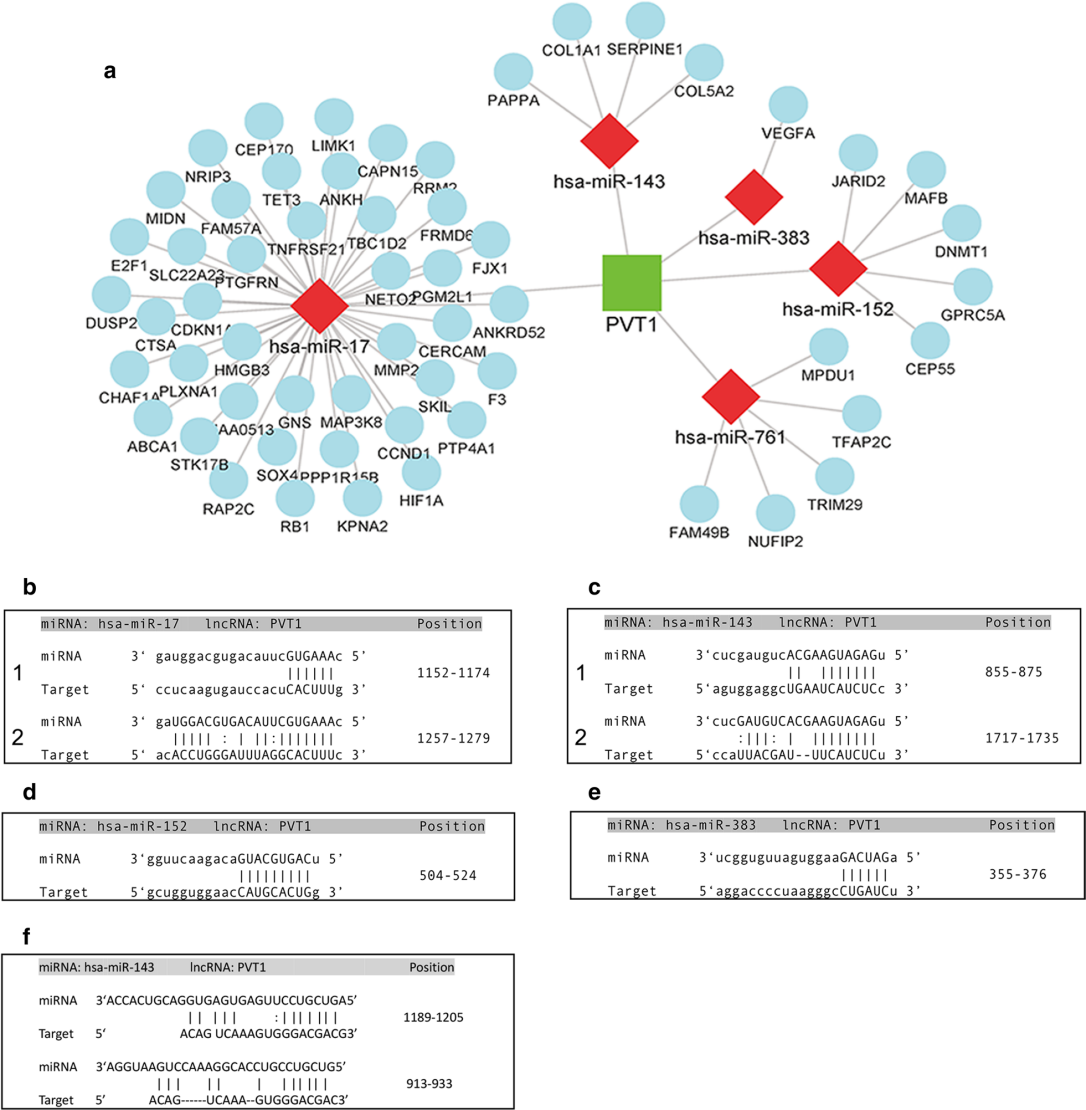
**a** PVT1 subnetwork. **b** Predicted interaction of hsa-miR-17 and PVT1 (by miRanda). **c** Predicted interaction of hsa-miR-143 and PVT1 (by miRanda). **d** Predicted interaction of hsa-miR-152 and PVT1 (by miRanda). **e** Predicted interaction of hsa-miR-383 and PVT1 (by miRanda)

PVT1 was the core lncRNA, and had the highest degree in the network. A previous study found that knockdown of lncRNA PVT1 reversed the murine angiotensin II-induced AAA associated alterations, such as attenuation of aortic diameter dilation and marked adventitial thickening [11]. To explore the regulatory mechanism of PVT1 in IA, we focused on the PVT1 subnetwork. The components of the PVT1 subnetwork are shown in Fig. 6a. The predicted or validated binding sites of hsa-miR-17, hsa-miR-143, hsa-miR-152, hsa-miR-761 and hsa-miR-383 with PVT1 are shown (Fig. 6b–e).

### Interaction between lncRNA and mRNA in the ceRNA network

**Fig. 7:**
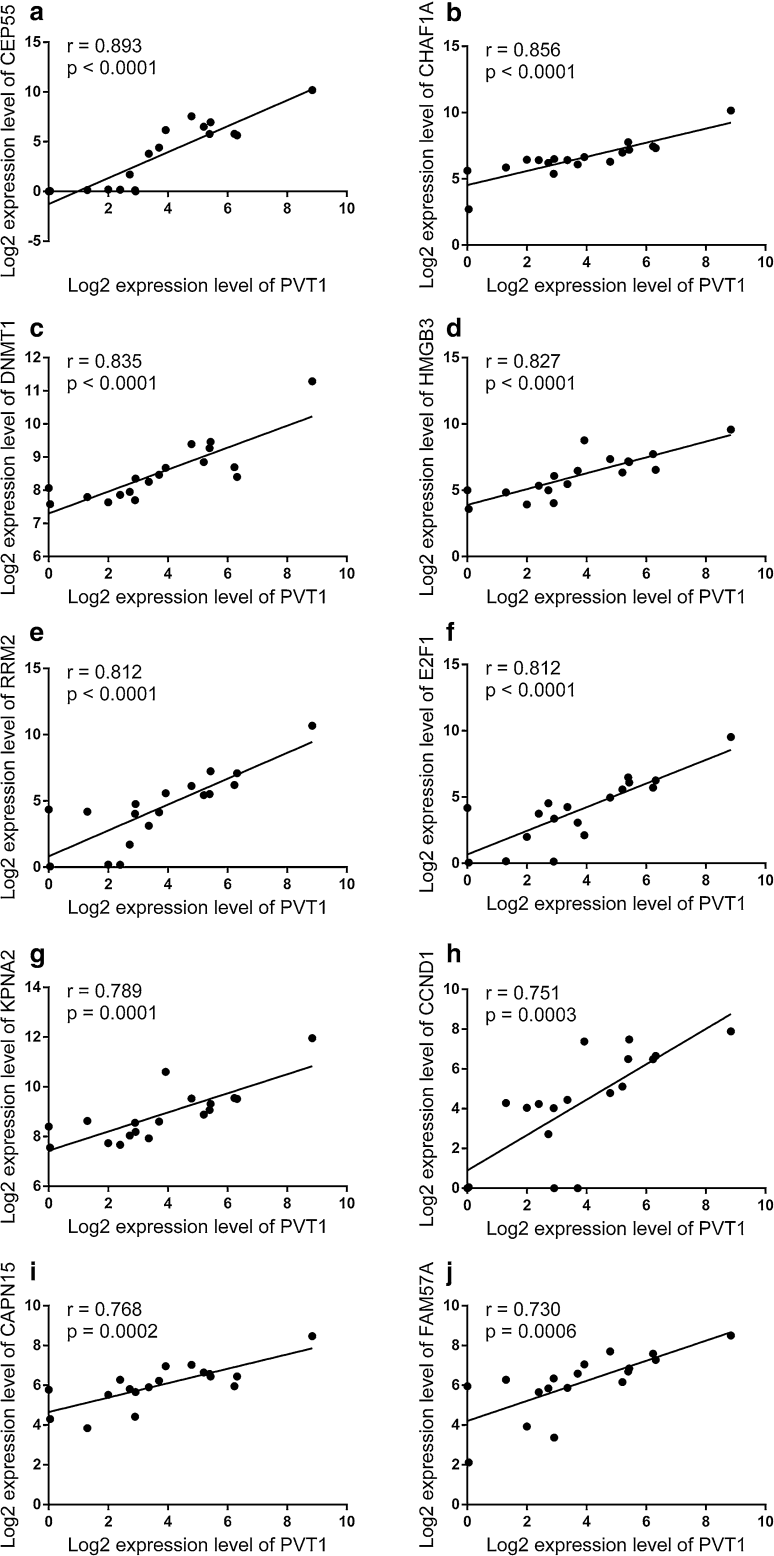
Pearson’s correlation between the expression levels of PVT1 and mRNAs in its subnetwork. The r represents correlation coefficient

LncRNA can interact with mRNAs indirectly following post-transcriptional regulatory mechanisms. We analyzed the correlation between PVT1 and mRNA’s expression levels in its subnetwork, which showed that there were strong positive correlations. There were 52 mRNAs interacting indirectly with PVT1. The correlation coefficients between 38.5% (20/52) mRNAs and PVT1 were more than 0.6 (Additional file 3: Table S3), and the correlation coefficients between 90.4% (47/52) mRNAs and PVT1 were more than 0.3 (Additional file 3: Table S3). Scatter plots of the top 10 mRNAs with the highest correlation coefficients are shown in Fig. 7.

### The non-coding RNAs-involved PI3K-AKT signaling pathway

**Fig. 8:**
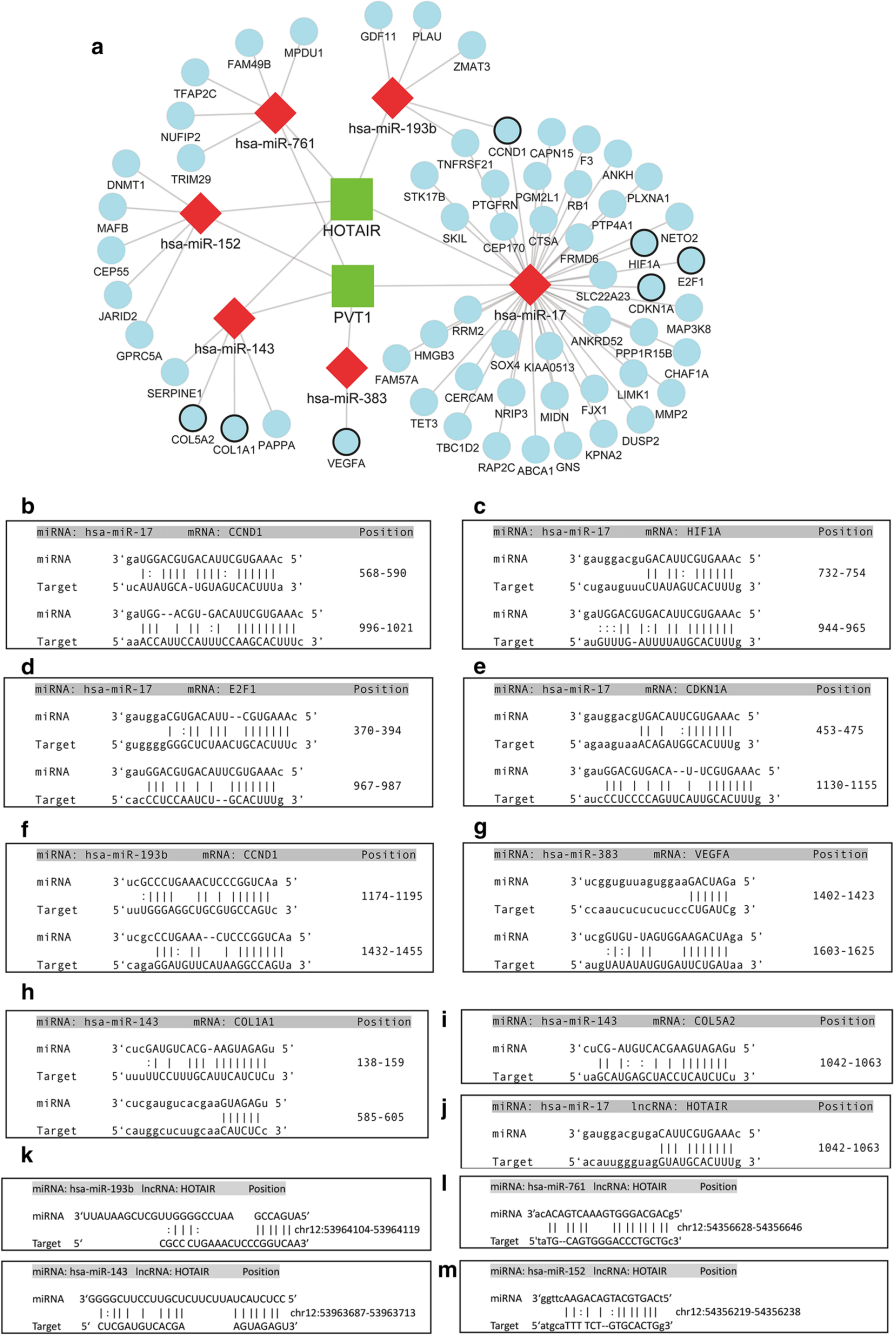
**a** Subnetwork of PVT1 and HOTAIR. The mRNA circles with dark outline are involved in PI3K-Akt signaling pathway. **b** Predicted interaction of hsa-miR-17 and CCND1 (by miRanda). **c** Predicted interaction of hsa-miR-17 and HIF1A (by miRanda). **d** Predicted interaction of hsa-miR-17 and E2F1 (by miRanda). **e** Predicted interaction of hsa-miR-17 and CDKN1A (by miRanda). **f** Predicted interaction of hsa-miR-193b and CCND1 (by miRanda). **g** Predicted interaction of hsa-miR-383 and VEGFA (by miRanda). **h** Predicted interaction of hsa-miR-143 and COL1A1 (by miRanda). **i** Predicted interaction of hsa-miR-143 and COL5A2 (by miRanda). **j** Predicted interaction of hsa-miR-17 and HOTAIR (by miRanda). **k** Predicted interaction of hsa-miR-193b, hsa-miR-143 and HOTAIR (by miRanda). **l** Predicted interaction of hsa-miR-761 and HOTAIR (by miRanda). **m** Predicted interaction of hsa-miR-152 and HOTAIR (by miRanda)

**Fig. 9:**
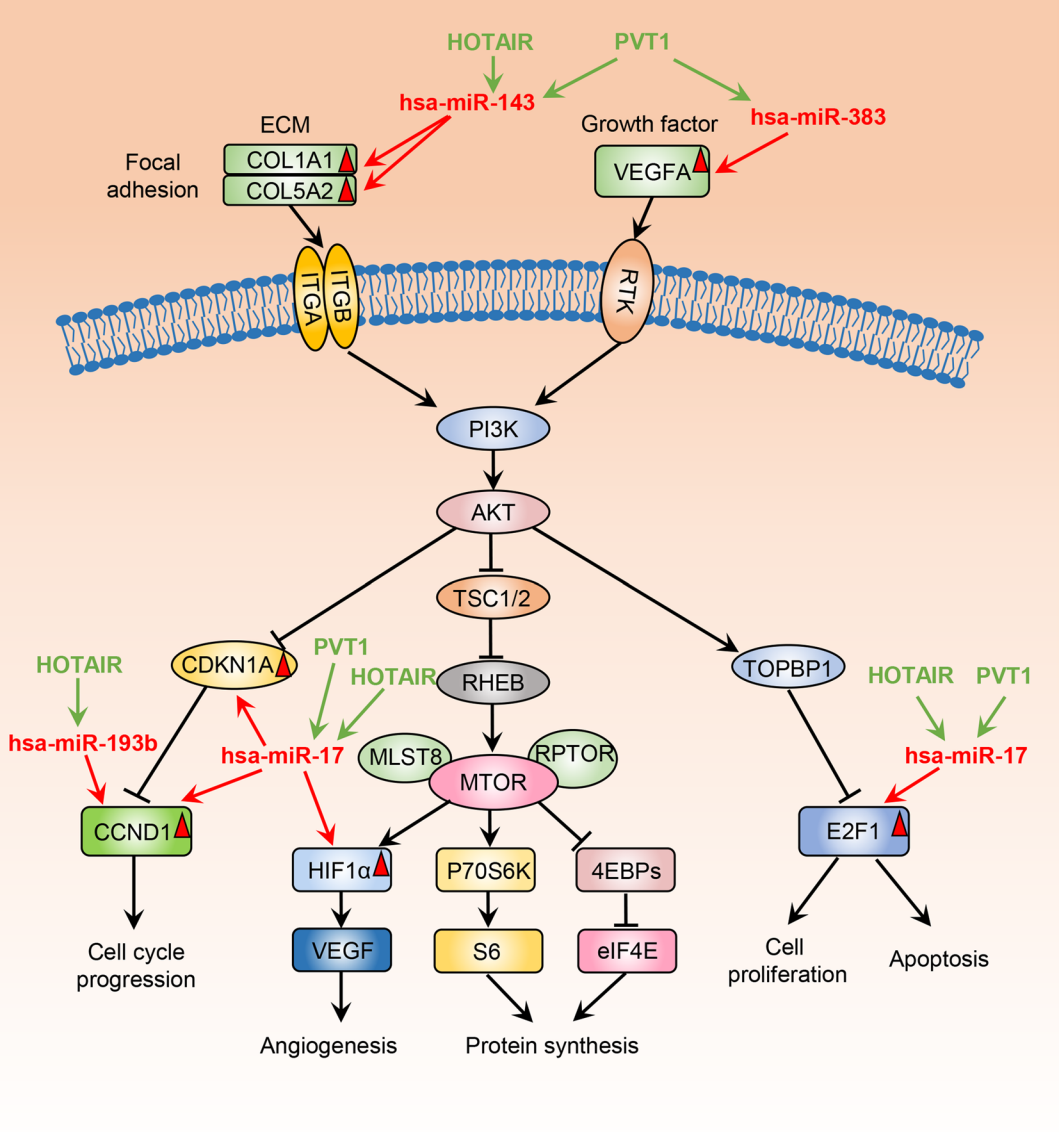
The ncRNAs involved the PI3K-Akt signaling pathway. The nodes labeled in red triangles were mRNAs in ceRNA network. The lncRNAs were labeled in green, and miRNA were labeled in red. The ncRNAs were annotated to PI3K-Akt signaling pathway according to previous studies and the predicted interactions in ceRNA network. The red and green arrows represented interactions between these RNAs

The KEGG pathway analysis revealed that the PI3K-AKT signaling pathway was significantly enriched, indicating that the PI3K-AKT signaling pathway could play an important role in IA formation. In addition, we found that several mRNAs in PVT1 subnetwork were curial genes in the PI3K-AKT signaling pathway, such as CCND1, HIF1A, E2F1, CDKN1A, VEGFA, COL1A1, and COL5A2. Moreover, these mRNAs were directly connected with hsa-miR-17, hsa-miR-152, hsa-miR-383, and hsa-miR-193b, and these miRNAs were connected with PVT1 and HOTAIR (Fig. 8a). hsa-miR-17, hsa-miR-143, hsa-miR-193b and hsa-miR-383 and their predicted or validated binding sites with these mRNAs were shown (Fig. 8b–i). hsa-miR-17, hsa-miR-143, hsa-miR-193b, hsa-miR-761, hsa-miR-152 and their predicted or validated binding sites with LncRNA HOTAIR was shown in Fig. 8j–m. To explore how these non-coding RNAs (ncRNAs) regulate the PI3K-AKT signaling pathway and further affect IA formation, we mapped these ncRNAs to the PI3K-AKT signaling pathway (Fig. 9). Regulation of E2F transcription factor 1 (E2F1) by topoisomerase 2-binding protein 1 (TOPBP1) involves the PI3K-AKT signaling pathway, which can mediate both cell proliferation and apoptosis [25]. Hypoxia inducible Factor 1 Subunit Alpha (HIF1A) is regulated by PI3K-AKT-MTOR signaling pathway, and further promotes angiogenesis [26]. CDKN1A and CCND1 can regulate cell cycle, which are also part of the PI3K-AKT signaling pathway [27]. VEGFA can bind to VEGFR1/2 and further activate PI3K-AKT signaling pathway [28].

## Discussion

LncRNA are a type of non-coding RNA with more than 200 bp transcript length. Various studies have found that lncRNAs play important roles in different types of diseases. Large amounts of lncRNAs have been annotated, however, the functions of them, especially in diseases, still need to be further explored. Many previous studies focused on ncRNA-mediated regulation of mRNAs, but recent studies have indicated that lncRNAs could regulate mRNAs via miRNAs and further form a regulatory ceRNA network. The ceRNA hypothesis refers that the lncRNAs and mRNAs compete to bind miRNAs and further regulate each other’s expression levels. Recently, various studies have focused on the functions of ceRNA networks in cardiac hypertrophy, type 2 diabetes mellitus, and various types of cancer [5–8]. However, the potential role of the ceRNA network in IA formation remains unclear.

In this study, all of the DEmiRNAs in the ceRNA network were downregulated in IAs relative to STAs. According to ceRNA theory, the down-regulated lncRNAs and mRNAs were excluded. Previous studies found that downregulation of miR-143/145 cluster could upregulate their targeted gene KLF5, and further promote proliferation and migration of VSMCs [29]. In our study, we found that miR-143 interacted with lncRNA PVT1 and HOTAIR, and that miR-143 could target COL1A1 and COL5A2 and further affect the PI3K-Akt signaling pathway. Thus, we may provide a potential axis PVT1/HOTAIR-miR-143-COL1A1/COL5A2 as an upstream regulator of the PI3K-Akt signaling pathway.

The PI3K-Akt signaling pathway is activated by various types of cellular stimuli or toxic insults, and regulates fundamental cellular functions such as transcription, translation, proliferation, growth, and survival. Dysregulation in the PI3K-Akt signaling pathway is implicated in various diseases such as cancer, type 2 diabetes mellitus, and IA [7, 30, 31]. Previous research reported that activation of PI3K-Akt signaling pathway promoted proliferation of vascular smooth muscle cells which were found to be involved in aortic aneurysms [32]. Inhibition of VEGF/PI3K/Akt signaling pathway mediated by miR-195 could suppress formation of abdominal aortic aneurysm [33]. In addition, the PI3K-Akt signaling pathway was less studied in IA formation. Sun et al. found that bone marrow mesenchymal stem cells-derived exosomes supressed the activation of the PI3k/Akt/NF-κB signaling pathway and maintained Th17/Treg balance, which could inhibite development of IA. Recently, various studies revealed that various lncRNAs affect the PI3K-Akt signaling pathway by targeting miRNAs, and further regulating mRNAs in the pathway [34, 35].

In our study, we found that PVT1 and HOTAIR were involved in the regulation of PI3K-Akt signaling pathway by targeting different miRNAs and regulating upstream/downstream effectors (COL1A1, COL5A2, VEGFA, CCND1, HIF1A, E2F1 and CDKN1A). Several members of collagen family, such as COL1A1, COL5A2, were overexpressed in IAs, which were consistent with previous studies [36, 37]. And these two genes were involved in collagen formation that could result in the ECM remodeling of intracranial blood vessels, which promotes formation of IA [36]. VEGFA, related to aSAH, was reported to be associated with thrombospondin and vascular endothelial growth factor [38]. A previous study revealed that HIF1A may be associated with rupture of human saccular IA wall [39]. Another research found that HIF1A was pivotal for the development of AAA [40]. HIF1A could upregulate expression levels of MMP-2 and MMP-9, and further promote aneurysmal progression [40]. Down-regulation of PVT1 could reduce proinflammatory cytokines, MMP-2 and MMP-9, increase TIMP-1, and further reverse angiotensin II-induced AAA-associated alterations in mice [11]. In this study, we annotated these ncRNAs to PI3K-Akt signaling pathway. These findings establish novel connections among lncRNAs, miRNAs, and mRNAs in PI3K-Akt signaling pathway, which play an important role in IA.

In this study, we also found that there were strong correlations between PVT1 expression and several genes’ expression levels (Fig. 7 and Additional file 3: Table S3), including DNMT1, E2F1 et al. Jin et al. reported that PVT1 could recruit DNMT1 via EZH2 to miR-18b-5p DNA promoter and inhibited the miR-18b-5p transcription via DNA methylation [41]. Previous study found that PVT1 could regulate E2F1 expression levels in tumor development [42]. These genes might also play roles in development of IA, which needs more verification in future research. The potential axis PVT1/HOTAIR-miR-143-COL1A1/COL5A2 needs further verification in future research, and the function of the axis in formation of IA needs to be explored.

## Conclusion

First, we identified differentially expressed lncRNAs, miRNAs and mRNAs between STAs and IAs. Functional enrichment analysis of differentially expressed mRNAs was performed. We further developed a lncRNA–miRNA–mRNA regulatory network. Then, the subnetworks of each core miRNAs were extracted and analyzed. Moreover, PVT1, the hub lncRNA in the ceRNA network, was reported to be related to aneurysm. The subnetwork of PVT1 was extracted and analyzed. Furthermore, the correlations of expression levels between PVT1 and mRNAs in its subnetwork were analyzed. In addition, we found that several mRNAs in the PVT1 subnetwork, including CCND1, HIF1A, E2F1, CDKN1A, VEGFA, COL1A1, and COL5A2, were an important part of the PI3K-Akt signaling pathway. We found that several ncRNAs were connected with these mRNAs. Then, we annotated these ncRNAs to the PI3K-Akt signaling pathway. This study may provide a comprehensive view of the underlying mechanisms of gene regulation and interaction in IA. Additionally, this study also revealed that ncRNAs involved in the PI3K-Akt signaling pathway could play an important role in IA formation.

## Supplementary Information

**Additional file 1: Table S1.** Interactions between lncRNA and miRNA in the ceRNA network.

**Additional file 2: Table S2.** Interactions between miRNA and mRNA in the ceRNA network.

**Additional file 3: Table S3.** Correlation analysis of the relationships between lncRNA PVT1 and mRNAs.

## Data Availability

The expression data analyzed in this study are available in GEO public repository (GSE66240).

## Abbreviations

IA: Intracranial aneurysm
miRNAs: MicroRNAs
lncRNA: Long noncoding RNA
DE: Differentially expressed
ncRNAs: Non-coding RNAs
STA: Superficial temporal artery
SAH: Subarachnoid hemorrhage
ceRNA: Competitive endogenous RNA
RMA: Robust multi-array average (RMA)
GO: Gene ontology
KEGG: Kyoto Encyclopedia of Genes and Genomes
AAA: Abdominal aortic aneurysm
E2F1: E2F transcription factor 1
TOPBP1: Topoisomerase 2-binding protein 1
