# Supplementary material for: Construction of competitive endogenous RNA network reveals regulatory role of long non-coding RNAs in intracranial aneurysm
Source: BMC Neurosci. 2021 Mar 9;22:15. doi: 10.1186/s12868-021-00622-7 (PMC7945298; doi:10.1186/s12868-021-00622-7)
Supplement: Supplementary file 1 — Additional file 1: Table S1. Interactions between lncRNA and miRNA in the ceRNA network. [file 12868_2021_622_MOESM1_ESM.docx]

**Table S1. Interactions between lncRNA and miRNA in the ceRNA network.**

| lncRNA | miRNA |
| --- | --- |
| GRIK1-AS1 | hsa-miR-383, hsa-miR-761 |
| HMGA1P4 | hsa-miR-143, hsa-miR-4770 |
| HOTAIR | hsa-miR-143, hsa-miR-152, hsa-miR-17, hsa-miR-193b, hsa-miR-4770, hsa-miR-761 |
| KCNQ1OT1 | hsa-miR-105, hsa-miR-143, hsa-miR-152, hsa-miR-17, hsa-miR-187, hsa-miR-193b, hsa-miR-4770, hsa-miR-761 |
| LINC00163 | hsa-miR-143, hsa-miR-193b, hsa-miR-4770, hsa-miR-761 |
| LINC00242 | hsa-miR-152, hsa-miR-761 |
| LINC00337 | hsa-miR-105, hsa-miR-17, hsa-miR-383 |
| LINC00460 | hsa-miR-143, hsa-miR-4770 |
| LINC00520 | hsa-miR-105, hsa-miR-17 |
| MEG3 | hsa-miR-105, hsa-miR-143, hsa-miR-17, hsa-miR-383 hsa-miR-4770, hsa-miR-761 |
| MIAT | hsa-miR-105, hsa-miR-143, hsa-miR-152, hsa-miR-17, hsa-miR-187 hsa-miR-4770, hsa-miR-761 |
| MIR155HG | hsa-miR-761 |
| MIR210HG | hsa-miR-105, hsa-miR-761 |
| MIR31HG | hsa-miR-193b, hsa-miR-761 |
| MTUS2-AS1 | hsa-miR-187 |
| NEAT1 | hsa-miR-105, hsa-miR-143, hsa-miR-152, hsa-miR-17, hsa-miR-193b, hsa-miR-383, hsa-miR-4770, hsa-miR-761 |
| PVT1 | hsa-miR-105, hsa-miR-143, hsa-miR-152, hsa-miR-17, hsa-miR-187, hsa-miR-383, hsa-miR-4770, hsa-miR-761 |
| SFTA1P | hsa-miR-143, hsa-miR-4770 |
| SNHG1 | hsa-miR-143, hsa-miR-383, hsa-miR-4770 |
| SNHG3 | hsa-miR-105, hsa-miR-152, hsa-miR-17, hsa-miR-761 |
| SPAG5-AS1 | hsa-miR-105, hsa-miR-17, hsa-miR-187, hsa-miR-761 |
| UCA1 | hsa-miR-143, hsa-miR-193b, hsa-miR-383, hsa-miR-4770, hsa-miR-761 |
