# Supplementary material for: Construction of competitive endogenous RNA network reveals regulatory role of long non-coding RNAs in intracranial aneurysm
Source: BMC Neurosci. 2021 Mar 9;22:15. doi: 10.1186/s12868-021-00622-7 (PMC7945298; doi:10.1186/s12868-021-00622-7)
Supplement: Supplementary file 2 — Additional file 2: Table S2. Interactions between miRNA and mRNA in the ceRNA network. [file 12868_2021_622_MOESM2_ESM.docx]

**Table S2. Interactions between miRNA and mRNA in the ceRNA network.**

| miRNA | mRNA |
| --- | --- |
| hsa-miR-143 | COL1A1, COL5A2, PAPPA, SERPINE1 |
| hsa-miR-152 | CEP55, DNMT1, GPRC5A, JARID2, MAFB |
| hsa-miR-17 | ABCA1, ANKH, ANKRD52, CAPN15, CCND1, CDKN1A, CEP170, CERCAM, CHAF1A, CTSA, DUSP2, E2F1, F3, FAM57A, FJX1, FRMD6, GNS, HIF1A, HMGB3, KIAA0513, KPNA2, LIMK1, MAP3K8, MIDN, MMP2, NETO2, NRIP3, PGM2L1, PLXNA1, PPP1R15B, PTGFRN, PTP4A1, RAP2C, RB1, RRM2, SKIL, SLC22A23, SOX4, STK17B, TBC1D2, TET3, TNFRSF21 |
| hsa-miR-193b | CCND1, GDF11, PLAU, TNFRSF21, ZMAT3 |
| hsa-miR-383 | VEGFA |
| hsa-miR-761 | FAM49B, MPDU1, NUFIP2, TFAP2C, TRIM29 |
