# Supplementary material for: Construction of competitive endogenous RNA network reveals regulatory role of long non-coding RNAs in intracranial aneurysm
Source: BMC Neurosci. 2021 Mar 9;22:15. doi: 10.1186/s12868-021-00622-7 (PMC7945298; doi:10.1186/s12868-021-00622-7)
Supplement: Supplementary file 3 — Additional file 3: Table S3. Correlation analysis of the relationships between lncRNA PVT1 and mRNAs. [file 12868_2021_622_MOESM3_ESM.docx]

**Table S3. Correlation analysis of the relationships between lncRNA PVT1 and mRNAs.**

| lncRNA | mRNA | R | P value |
| --- | --- | --- | --- |
| PVT1 | CEP55 | 0.893 | < 0.0001 |
| PVT1 | CHAF1A | 0.856 | < 0.0001 |
| PVT1 | DNMT1 | 0.835 | < 0.0001 |
| PVT1 | HMGB3 | 0.827 | < 0.0001 |
| PVT1 | RRM2 | 0.812 | < 0.0001 |
| PVT1 | E2F1 | 0.812 | < 0.0001 |
| PVT1 | KPNA2 | 0.789 | 0.0001 |
| PVT1 | CAPN15 | 0.768 | 0.0002 |
| PVT1 | CCND1 | 0.751 | 0.0003 |
| PVT1 | TFAP2C | 0.75 | 0.0003 |
| PVT1 | FAM57A | 0.730 | 0.0006 |
| PVT1 | FJX1 | 0.729 | 0.0006 |
| PVT1 | PTP4A1 | 0.726 | 0.0007 |
| PVT1 | NETO2 | 0.723 | 0.0007 |
| PVT1 | LIMK1 | 0.704 | 0.0011 |
| PVT1 | TBC1D2 | 0.698 | 0.0013 |
| PVT1 | RAP2C | 0.646 | 0.0038 |
| PVT1 | STK17B | 0.637 | 0.0045 |
| PVT1 | CTSA | 0.627 | 0.0053 |
| PVT1 | TNFRSF21 | 0.624 | 0.0056 |
| PVT1 | PTGFRN | 0.621 | 0.006 |
| PVT1 | TET3 | 0.577 | 0.012 |
| PVT1 | GPRC5A | 0.576 | 0.012 |
| PVT1 | VEGFA | 0.574 | 0.013 |
| PVT1 | DUSP2 | 0.564 | 0.0003 |
| PVT1 | NRIP3 | 0.555 | 0.017 |
| PVT1 | MIDN | 0.553 | 0.017 |
| PVT1 | HIF1A | 0.540 | 0.02 |
| PVT1 | RB1 | 0.536 | 0.022 |
| PVT1 | PPP1R15B | 0.535 | 0.022 |
| PVT1 | COL1A1 | 0.534 | 0.023 |
| PVT1 | JARID2 | 0.532 | 0.023 |
| PVT1 | PAPPA | 0.520 | 0.027 |
| PVT1 | F3 | 0.513 | 0.03 |
| PVT1 | SLC22A23 | 0.511 | 0.03 |
| PVT1 | ANKRD52 | 0.501 | 0.034 |
| PVT1 | SKIL | 0.495 | 0.037 |
| PVT1 | SERPINE1 | 0.470 | 0.049 |
| PVT1 | PGM2L1 | 0.457 | 0.056 |
| PVT1 | CEP170 | 0.452 | 0.06 |
| PVT1 | KIAA0513 | 0.451 | 0.06 |
| PVT1 | CDKN1A | 0.431 | 0.074 |
| PVT1 | COL5A2 | 0.425 | 0.079 |
| PVT1 | NUFIP2 | 0.402 | 0.0047 |
| PVT1 | MPDU1 | 0.396 | 0.0052 |
| PVT1 | SOX4 | 0.393 | 0.107 |
| PVT1 | ABCA1 | 0.387 | 0.113 |
| PVT1 | MAP3K8 | 0.380 | 0.12 |
| PVT1 | MAFB | 0.368 | 0.133 |
| PVT1 | PLXNA1 | 0.358 | 0.144 |
| PVT1 | ANKH | 0.312 | 0.208 |
| PVT1 | FAM49B | 0.304 | 0.0177 |
| PVT1 | TRIM29 | 0.07 | 0.29 |
| PVT1 | CERCAM | 0.069 | 0.292 |
| PVT1 | GNS | 0.047 | 0.386 |
| PVT1 | FRMD6 | 0.014 | 0.644 |
| PVT1 | MMP2 | 0.008 | 0.718 |
